# High level of αB-crystallin contributes to the progression of osteosarcoma

**DOI:** 10.18632/oncotarget.6928

**Published:** 2016-01-17

**Authors:** Qing-Ming Shi, Jun Luo, Kai Wu, Ming Yin, Yu-Rong Gu, Xi-Gao Cheng

**Affiliations:** ^1^ First Department of Orthopedic Surgery, The Second Affiliated Hospital of Nanchang University, Nanchang, Jiangxi, PR China

**Keywords:** osteosarcoma, αB-Crystallin, invasion, MMP-9, prognosis

## Abstract

Accumulating evidences indicate the elevated expression of αB-Crystallin (Cryab) is implicated in tumorigenesis. However, the expression and biologic role of Cryab in osteosarcoma (OS) are still unknown. In this study, we showed that Cryab expression was elevated in OS tissues and cell lines, and down-regulation of Cryab in MG-63 and U-2OS cells led to a decline in the cells’ aggressiveness, and reduced secretion of matrix metalloproteinase-9 (MMP-9) *in vitro,* and lower metastasis potential *in vivo*. Further study indicated that the Cryab expression was positively associated with the activity of ERK1/2 which is responsible for the cells’ aggressiveness and MMP-9 secretion. Clinically, our data confirmed that the high level of Cryab was associated with shorten survival and tumor recurrence for the postoperative OS patients. Together, our results indicate that high level of Cryab is a new adverse outcomes marker for OS patients and may be used as a new therapeutic target.

## INTRODUCTION

Osteosarcoma (OS) is the most frequent malignant bone tumor in children and young people [1]. Although the overall survival of patients with high-grade OS has been markedly improved over the past decades [2], the recurrence for this disease still occurs in approximately 30 - 40 percent of patients with non-metastatic OS, furthermore, almost 80 percent of the OS patients with metastasis at the time of diagnosis will recur [3, 4]. Generally, the standard multimodal therapy failure for OS is associated with a very poor prognosis [5, 6]. Therefore, it is very important and meaningful to screen for novel molecular markers and deepen our understanding of the molecular mechanisms linked to OS metastasis and invasion.

αB-crystallin (Cryab), a human small heat-shock proteins (sHsp), was first identified as one of the structure member of the vertebrate eye lens. Cryab presents with high levels in the lens, while low in other tissues including the cardiac and skeletal muscle, kidney, and brain [7]. The functions of Cryab have been found to be involved in a variety of processes including cytoskeletal assembly, remodeling and stabilization, apoptosis inhibition, and modulation of membrane fluidity [7]. In recent years, the increasing expression of Cryab have been identified to be related to a number of pathologies including cancer, with elevated expression of Cryab observed in several cancers, for example, a high level of Cryab has been found to be a prognostic marker in breast, renal, thyroid, nasopharyngeal, hepatocellular and lung cancers [8-11]. Furthermore, the up-regulation of Cryab expression seems to be associated with oncogenic transformation in basal-like breast cancer, and induces hepatocellular carcinoma cells epithelial-mesenchymal transition [9], In addition, the high level of Cryab was reported to be associated with cell apoptosis resistance, enhance cell survival under the oncogenic stress, growth factor starvation, chemotherapy, and other cellular stressors [12]. Given the key role of Cryab in human cancers development, further study of the role and mechanism of Cryab in OS is urgently needed.

The purpose of this experiment was to check the Cryab expression in OS cells, and in OS and adjacent nontumorous tissues, as well as assessing the role and mechanisms of Cryab in biological behaviours of OS cell lines through Cryab interference and cDNA transfection. Finally, the relationships between the expression of Cryab and clinical features and survival in OS patients were investigated.

## RESULTS

### Cryab is elevated expression in OS tissues

**Figure 1:**
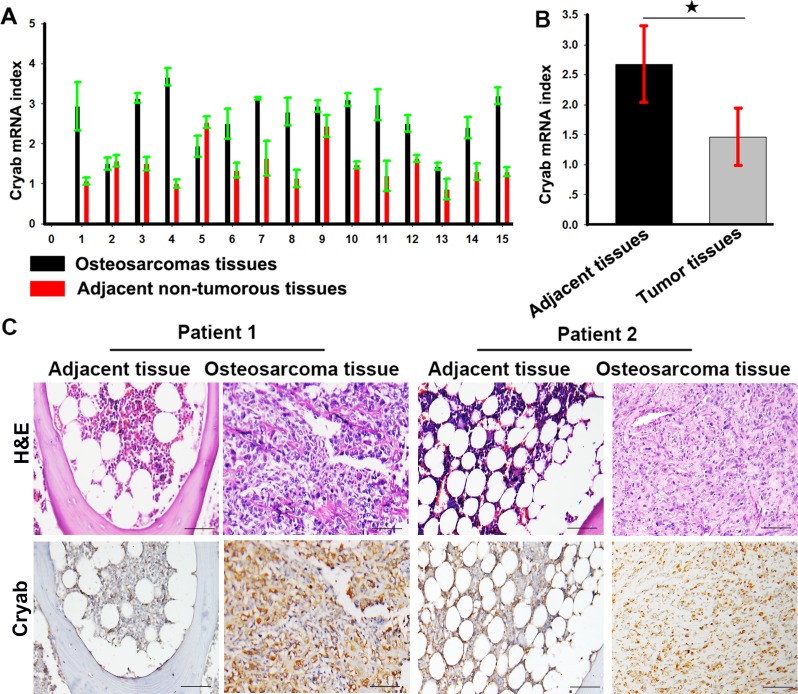
Cryab is elevated expression in OS tissues **A.** The expression of Cryab mRNA in OS samples and the adjacent nontumorous samples; **B.** a histogram showed Cryab mRNA in OS samples and the adjacent nontumorous samples (*p* < 0.05); **C.** Hematoxylin-eosin staining and immunostaining of Cryab in tumor and the adjacent nontumorous samples (bar = 200μm).

Expression of Cryab was examined by real time-PCR (qRT-PCR) in OS and adjacent nontumorous tissues. Low expression of Cryab was detected in nontumorous tissues compared with OS tissues. As shown in Figure 1A and 1B, the mean ΔCt value of Cryab expression was 2.68 ± 0.64 and varied significantly in OS samples (range 1.43 - 3.67), while mean expression level was only 1.46 ± 0.47 in adjacent nontumorous samples (range 0.87 - 2.53). The difference in Cryab expression between OS and adjacent nontumorous samples was statistically significant (*p* < 0.01). Immunohistochemistry also showed a high level of Cryab in OS samples compared with the adjacent nontumorous tissues (Figure 1C).

### Cryab promotes metastasis and invasion of OS *in vitro* and *in vivo*

**Figure 2:**
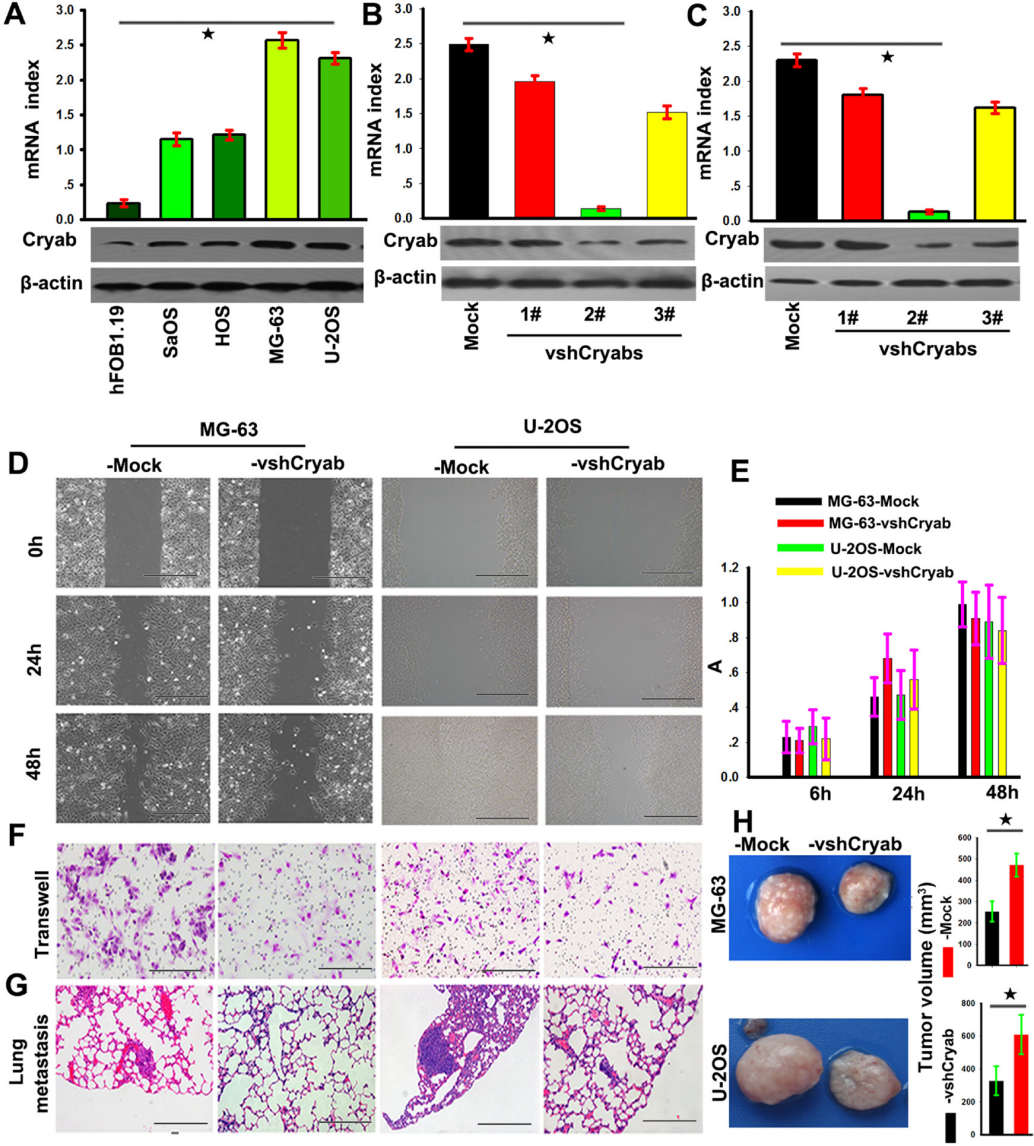
Cryab promoted the invasion and metastasis of OS cells *in vitro* and *vivo.* **A.** RT-PCR and immunoblotting analyzed the expression of Cryab in hFOB1.19, MG-63, U-2OS, Saos and HOS cell lines (*p* < 0.05); (B and C) The expression of Cryab were regulated in MG-63 and U-2OS cells by RNA interference; **D.** The wound-healing assay revealed that an evident delay in the wound closure rate of MG-63/U-2OS-vshRNA-Cryab cells was found at 24 and 48h, compared with MG-63/U-2OS-Mock cells (bar = 100 μm); **E.** The down-regulation of Cryab has no influence on cell proliferation (*p* > 0.05); **F.** Matrigel invasion assays showed that down-regulation of Cryab was accompanied by a descend invasion of OS cells (bar = 100μm); **G.** Serial sections from mouse lung showed the metastasis ability of cancer cells expressing different Cryab (bar = 50μm); **H.** The tumor volume of cancer cells expressing different level of Cryab.

To explore the biological roles of Cryab in OS, we firstly evaluated the Cryab expression in *hFOB1.19,* Saos, HOS, U-2OS and MG-63 cell lines, and found that Cryab expression in OS cells was higher than that in *hFOB1.19* cells at the level of mRNA and protein (*p* < 0.05). Then, MG-63 and U-2OS cells were transfected with pGPU6-GFP-vshRNA-Cryabs. Of three vshRNA-Cryabs tested, #2 was testified by qRT-PCR and western blotting for the most efficient interference of Cryab (Figure 2A, 2B and 2C) and chosen for further study. The scratch assay presented that a clear delay in the wound closure rate of shRNA-Cryab-MG-63/U-2OS cells was found at 24 h, compared with MG-63/U-2OS-Mock cells (Figure 2D). The transwell assay showed that the down-regulation of Cryab expression inhibited the invasiveness of MG-63 and U-2OS cells (Figure 2F). However, the OS cells proliferation were not inhibited by the interference of Cryab (*p* > 0.05, Figure 2E). *In vivo* analysis showed that the tumor volumes of in the shRNA-Cryab-MG-63 or U-2OS group were smaller compared to their controls (264±35 vs. 482±64 and 358±56 vs. 613±109, *p* < 0.05, Figure 2H), and the incidences of lung metastasis were 40% in the shRNA-Cryab-MG-63/U-2OS groups compared to 100% in the control groups (Figure 2G and Supplementary Figure 1). These results indicated that up-regulation of Cryab was along with the increased metastatic potential of OS cells.

### High level of cryab induces OS progression *via* up-regulation of MMP9 expression through ERK1/2 signaling

**Figure 3:**
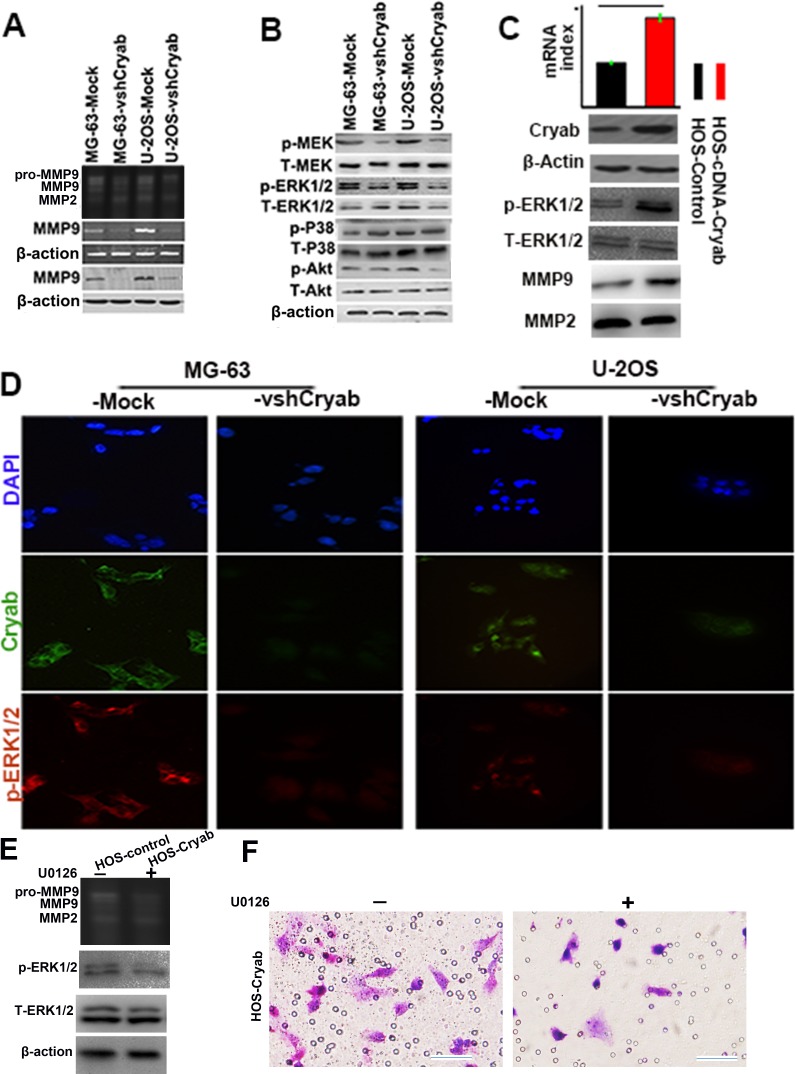
High level of Cryab induces OS progression *via* up-regulation of MMP9 expression through ERK1/2 signaling **A.** The secretion and expression of MMP9 were inhibited by the Cryab specific RNA interference in OS cells; **B.** The phosphorylation level of MEK and ERK1/2 were reduced significantly in MG-63/U-2OS-vshRNA-Cryab cells, compared with MG-63/U-2OS-Mock cells; **C.** The level of ERK1/2 activity was elevated, and the expression of MMP9 were up-regulated by Cryab cDNA transfection in HOS cells; **D.** Immunofluorescent staining showed that the expression of Cryab was in line with the activation of ERK1/2; **E.** The inhibition of ERK1/2 phosphorylation by U0126 was associated with the inhibition of secretion of MMP9 and OS cells invasion (bar = 200μm).

Matrix metalloproteinases (MMPs) have always been with cancer cell invasion and metastasis [13]. Here, we tried to determine the effect of Cryab on the secretion and expression of MMP-2 and 9. Gelatinase activity assays displayed that the expression of Cryab positively associated with the secretion and expression of MMP-9, but not MMP-2 (Figure 3A). According to a previous report concerning the mechanisms of Cryab promotion of cancer progression, the amplitude of phosphorylation of MEK, p38, ERK1/2 and Akt in shRNA-Cryab-MG-63/U-2OS cells were compared to their corresponding matched cells. As shown in Figure 3A, the elevated levels of MEK and ERK1/2 were detected in both MG-63/U-2OS-Mock cells compared with the shRNA-Cryab-MG-63/U-2OS cells, while the p38 phosphorylation was not changed (Figure 3B). Immunofluorescent staining showed that the interference of Cryab in shRNA-Cryab-MG-63/U-2OS cells down-regulated the level of phosphorylation of ERK1/2 compared with the Mock-MG-63/U-2OS cells (Figure 3D).

In order to identify the signaling pathways that might contribute to the observed phenotypic changes, we up-regulated the Cryab expression in HOS cells by transfecting Cryab cDNA, we found that the phosphorylation of ERK was much higher in HOS-cDNA-Cryab cells, and increased the secretion of MMP9 compared with HOS-Mock cells (Figure 3C). Furthermore, U0126-exposure resulted in an evident inhibition of MMP-9 secretion and cells invasion (Figure 3E). These results indicate that ERK1/2 hyperactivation appears to be pivotal for the appeared Cryab-mediated phenotypic characteristics of OS cells.

### Expression of Cryab was positively associated with malignant phenotypes of OS by immunohistochemistry

**Figure 4:**
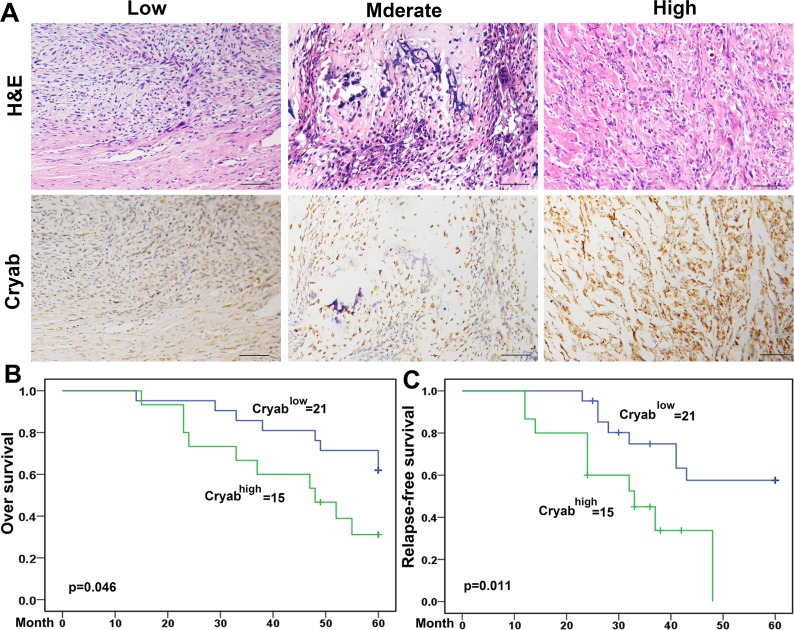
Cryab overexpression was associated with the poor prognosis of OS Patients **A.** Immunoreactivity of Cryab protein was located in the cytoplasm of the carcinoma cells (bar = 200μm); **B.** Prognostic significance assessed by Kaplan-Meier survival estimates and log-rank tests. Comparisons of OS and RFS by Cryab.

Positive Cryab staining was detected in the cytoplasm of tumor cells and showed substantial heterogeneity in the dissimilar tumor specimens (Figure 4A). Cryab^high^ accounted for 41.67% (15/36) of the total number of patients. Agree with a preceding report [20], patients with high Cryab expression increased chance to exhibit aggressive features. As shown in Table 1, Cryab^high^ was significantly correlated with high Enneking stage (*p* = 0.001), a poor response to chemotherapy (*p* = 0.032) and metastasis (*p* = 0.019) compared to those patients with low expression of Cryab. However, additional clinical features, containing age, sex, and site of primary tumor were not significantly relevant to the Cryab expression.

**Table 1:** Correlation between Cryab expression and OS clinicopthological parameters

		Result of immunostaining (No. of patients)			
Parameter		Cryab-negative (n = 21/36)	Cryab-positive (n = 15/36)		*p*
Age (yrs)					
	<19	13	8		
	≥19	8	7	1.000	
Gender					
	Male	12	11		
	Female	9	4	0.054	
Site of primary tumor †					
	Femur	14	8		
	Tibia	3	3		
	Humerus	3	2		
	Pelvis	0	1		
	Other	1	1	0.103	
Histologic differentiation ††					
	Osteoblastic	10	13		
	Chondroblastic	2	2		
	Fibroblastic	5	0		
	Telangiectatic	3	0		
	Other	1	0	0.049	
Enneking stage ‡					
	I	2	0		
	IIA	6	1		
	IIB	12	11		
	III	1	3	0.001^a^	
Response to chemotherapy *					
	Good	15	7		
	Poor	4	8		
	NA	2	0	0.032	
Metastasis					
	Negative	14	4		
	Positive	7	11	0.019	

Note:

† Femur *vs.* Tibia/ Humerus/Pelvis/Other;

†† Osteoblastic *vs.* Chondroblastic/ Fibroblastic/ Telangiectatic/Other;

‡ I/IIA *vs.* IIB/III;

* good *vs.* poor/NA;

a Fisher's exact probability.

### High level of Cryab was associated with poor prognosis of OS patients

Up to the final follow-up, the 5-year overall survival and relapse-free survival (RFS) in the whole population were 69.44%, and 62.35%, respectively. The 2- and 5-year overall survival in the Cryab^low^ group was apparently higher than that in the Cryab^high^ group (Figure 4B). The 2- and 5-year RFS in the Cryab^low^ group were apparently higher than those in the Cryab^high^ group (Figure 4C), indicating that Cryab expression predicts an unfavorable prognosis for patients with osteosarcoma. Univariate analysis showed that overexpression of Cryab, high Enneking stage, poor response to chemotherapy, and lymphatic metastasis were predictors of overall survival and RFS. Additional features containing age and sex had no prognostic significance for overall survival or RFS (Table 2).

**Table 2:** Univariate analysis of factors associated with OS survival and recurrence

Variables	Overall survival		RFS	
	Hazard ratio (95% CI)	*p*value	Hazard ratio (95% CI)	*p*value
Sex (Male *vs.* Female)	1.660 (0.590-4.670)	0.337	1.458(0.519-4.096)	0.463
Age (years) (<19 *vs.* ≥19)	1.009 (0.983-1.037)	0.506	1.012 (0.986-1.040)	0.381
Enneking stage (I/IIA *vs*. IIB/III)	3.276 (1.157-9.219)	0.017	3.225 (1.128-9.224)	0.029
Site of primary tumor (Femur *vs*. other)	0.977(0.378-8.2022.524)	0.962	1.077 (0.417-2.784)	0.879
Response to chemotherapy (good *vs*. poor/NA)	3.029 (1.125-8.153)	0.023	3.324(1.471-7.531)	0.004
Metastasis (negative *vs.* positive)	11.062 (1.463-83.627)	0.001	36.548 (4.632-288.356)	0.001
Cryab^low^ *vs.*Cryab^high^	2.772(1.085-7.083)	0.033	3.719 (1.316-10.510)	0.013

**Abbreviations and Note:** OS, Osteosarcoma; RFS, relapse-free survival; 95%CI, 95% confidence interval; HR, Hazard ratio; Cox proportional hazards regression model.

## DISCUSSION

In this study, we identified that the expression of Cryab was markedly increased in OS tissues compared with adjacent non-tumor tissues, and the high levels of Cryab were involved in invasion and metastasis of OS both *in vitro* and *in vivo.* Furthermore, we also confirmed that OS cell lines expressing high levels of Cryab had characteristic of increasing secretion of MMP9. The proliferation of OS cells did not varied by Cryab *in vitro*, however, the tumor volumes were large in groups with high Cryab expression, which might due to the difference in angiogenesis which is central for tumor growth [14-16]. Clinically, we found that the elevated expression of Cryab correlates with poor survival and with disease recurrence of OS patients in the clinical setting. The above results favor the notion that Cryab makes a substantial contribution to tumor cell invasion and metastasis.

Numerous previous clinical studies documented that the high levels of Cryab endowed primary tumors with metastatic capacity [17], however, in head and neck cancer, an absence of Cryab has been reported to be a powerful marker for poor prognosis [10]. Our results indicate that high level of Cryab boosts tumor progression, which was supported by several lines of evidence. Firstly, the expression of Cryab is positively associated with metastatic potential of OS cells, and forced down-regulation of Cryab correspondingly reduces the capacity for tumor metastasis and invasion both *in vitro* and *in vivo*. Second, we show that high expression of Cryab is associated with MMP-9 expression and secretion in OS cells. Lastly, we discover that elevated expression of Cryab occurs more frequently in various OS with poor prognosis-associated clinical variables, including high tumor staging and poor differentiation. Importantly, we also show that elevated expression of Cryab is correlated with poor survival and early disease recurrence in a cohort of OS patients. Thus, we concluded that high level of Cryab promoted OS progression.

Recently, Huang *et al*. reported that the elevated expression Cryab can induce hyperactivity of the ERK1/2 signaling pathway by forming a complex with 14-3-3ζ in human hepatocellular carcinoma [9], and Moyano *et al.* confirmed that high level of Cryab constitutively activates the MAPK kinase/ERK1/2 (MEK/ERK1/2) pathway in human basal-like breast tumors [8]. In line with previous reports, we also found that elevated expression of Cryab induces the activity MEK/ERK1/2 signaling in OS cells by employing RNA interference. Importantly, we show that the inhibition of ERK1/2 activity by U0126 led to reversal of the cancer phenotype conferred by high level of Cryab. While another report argued that Cryab inhibits the MEK/ERK pathway in rabbit lens cells [18], it is not difficult to conclude that high level of Cryab can induce MEK/ERK signaling which is necessary for the expression of MMP-9 in OS cells.

Taken together, our results indicate that the high level of Cryab is a new adverse outcomes marker for OS patients and may be used as a new therapeutic target.

## MATERIALS AND METHODS

### Cell lines and plasmids

An hFOB1.19 (a human osteoblastic cell line) and four OS cell lines, including MG-63, U-2OS, Saos and HOS (acquired from the Cell bank of Chinese Academy, Shanghai, China), were conventionally cultured in RPMI 1640 (Invitrogen, Paisley, UK) supplied with 10 percent heat-inactivated fetal calf serum (FCS), 1% L-glutamine, and penicillin/streptomycin (10,000 U/mL and 10,000 μg/mL), and kept at 37°C in a humidified incubator under 5% CO2. The pGPU6-GFP-vshRNA-Cryabs and pGMLV-Cryab was constructed by genomeditech biological company (Shanghai, China).

### Patients and follow-up

The data of OS patients who underwent surgical treatment at the Second Affiliated Hospital of Nanchang University between January 2006 and December 2012 were summarized and reexamined. The pathological diagnosis was verified by two pathologists. OS patients who were still in clinical treatment or who were kept in touch by conventional telephone contact were gathered in our study. The frozen specimens were conserved by soaking the tissue samples in liquid nitrogen offhandedly after surgical excision, and then storage at −80°C. The samples that were estimated by pathologists were employed for the qRT-PCR and western blot test. Paraffin-embedded osteosarcomas samples were conventionally prepared by the pathologist and kept in the department of pathology of our hospital. Written agreement from patients or their guardians was obtained and approved by the Nanchang University Ethics Committee. Clinicopathologic features of the present series of OS patients are shown in Table 1.

### RNA extraction and qRT-PCR

Fifteen OS and their correspondent non-tumorous specimens were employed to detect the Cryab expression, and the RNAs were extracted routinely according to the description elsewhere [4, 19]. The DNA amplification and revelation were accomplished by applying the ABI PRISM 7900 Sequence Detection System (Applied Biosystems, Foster City, CA). β-action was availed as an internal reference. Primers for Cryab were: 5′-CTTTGACCAGTTCTTCGGAG-3′ and 5′-TCCGTGTTCAGCTGCTGGTA-3′; and β-action: 5′-AGAGCTACGAGCTGCCTGAC-3′ and 5′-AGCACTGTGTTGGCGTACAG-3′. The PCR amplification were in accordance with the following steps: after denaturation at 94°C for 10 min, then following by 40 cycles of denaturation at 94°C for 20s, annealing at 59°C for 30s, and extension at 72°C for 60s. The relative value of Cryab mRNA was analyzed according to the means of relative cycle threshold (Ct) [20]. This test was repeated three times.

### Immunoblotting and immunofluorescence

Thirty mg protein extracted from there OS cell lines and samples were used for immunoblotting as described elsewhere [21, 22]. All the antibodies employed in our experiment were displayed in Supplementary Table 1. β-action (1:1000, cell signaling technology) was used as an internal reference. This test was repeated in triplicate. Immunofluorescence was performed as described elsewhere. Nuclei were counterstained with 4,6-diamidino-2-phenylindole (DAPI, Vector Laboratories, Burlingame, CA). All the antibodies employed in our experiment were displayed in Supplementary Table 1.

### MTT assay, cell migration, and Matrigel™ invasion assays

Two hundred μL cells were loaded into a 96-well plate (2000 cells/well), and cultured for 24, 48, or 72 h, and then 20 μL MTT solution was appended at the stipulated time and hatched for an additional 4 h. Subsequently, the medium was replaced by 150μl DMSO with 200 μL RPMI 1640, and the 96-well plate were rocked for 10 min. Absorbance was measured at 490 nm to test the amount of viable cells in each well. This test was repeated in triplicate.

The scratch assay was applied to appraise cells motility ability. When the cultured cells blended up to 80%-90% in 24-well plates, a scratch was maked by a plastic pipette tip across the cell surface. The remanent cells were cleaned triply to elute cell rubbish and incubated at 37°C with serum-free medium. At the certain time points, the scratching front were photographed and then compared.

The cell invasion assays were carried out in accordance with the previous reports [23]. This experiment was performed in triplicate.

### Gelatin zymography

Gelatin zymography assay was performed as described [18, 24].

### Metastasis assays *in vivo*

The OS cells (Including 2.0 × 10^6^ shRNA-Cryab-MG-63 or U-2OS cells and shRNA-Mock-MG-63 or U-2OS cells) were suspended in 100 mL serum-free RPMI 1640 medium with Matrigel™ (BD Biosciences) (1:1), and then injected into the flank of nude mice (s.c.). The implanted tumors emerged at the injection site 8 d later. The implanted tumor volume (V) was reckoned according to the formula: V = 0.5 × L × (S)^2^, in which L and S are the largest and smallest perpendicular tumor diameters, respectively, Mice were sacrificed at 21 days after injection as previous reports. Serial lung sections were used to calculate the total number of lung metastases under the microscope as previously report [5, 25]. Each group consisted of 6 mice.

### Construction of tissue microarrays and immunohistochemistry

Tissue microarrays (TMA) were constructed by Shanghai Biochip Co. Ltd, Shanghai as reported elsewhere. Briefly, the histologically by hematoxylin and eosin staining sections from the all OS patients was reviewed by pathologists, and then the paraffin blocks were pre-stamped with the representative areas. The 1-mm-diameter cylinder tissues from two different areas in the araffin blocks near the non-cancerous border (named intratumor and peritumor, respectively; A total of 4 punches) were contained in each group, together with different compares, to ensure reproducibility and homogenous staining of the slides. Thus, 2 different TMA blocks were constructed, each containing 72 cylinders.

Polyclonal rabbit anti-human Cryab (1:200; Novus Biologicals, Cambridge, UK) was employed to determine the Cryab protein expression. Images were captured by the Leica QWin Plus v3 software. The intensity of staining was assessed as described. The two levels of Cryab intensity of were classified according to the mean area of positive staining, and the cutoff value was 50% of tumor section, the ≥50 percent was positive, and negative was < 50 percent.

## SUPPLEMENTARY MATERIAL FIGURE AND TABLE


