# Supplementary material for: High level of αB-crystallin contributes to the progression of osteosarcoma
Source: Oncotarget. 2016 Jan 17;7(8):9007–16. doi: 10.18632/oncotarget.6928 (PMC4891021; doi:10.18632/oncotarget.6928)
Supplement: Supplementary file 1 [file oncotarget-07-9007-s001.pdf]

High level of αB-crystallin contributes to the progression of osteosarcoma

Supplementary Material

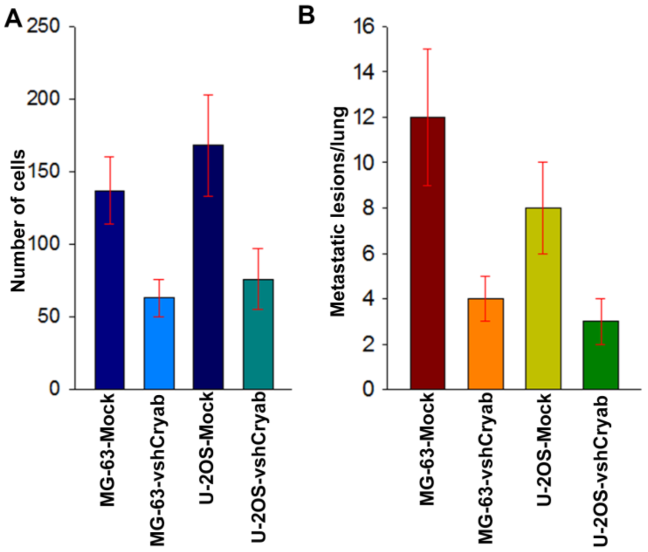

Supplemental Figure 1: A. The histogram of invasive cells, **B.** the histogram of metastasic lesions.

Table S1. The primary antibodies for western blot, IHC and IF

Antibody	Concentration for WB	Concentration for IHC	Concentration for IF	Concentration for co-IP	Specificity	Company
Cryab	1:3000	1:200	1:100	1:1000	Rat polyclonal	Novus Biologicals
. β-action	1:1000	/	/	/	Rabbit polyclonal	Cell Signaling Technology
AKT	1:1000	/	/	/	Rabbit polyclonal	Cell Signaling Technology
p-AKT^{s473}	1:1000	/	1:100	/	Rabbit polyclonal	Cell Signaling Technology
ERK1/2	1:1000	/	/	/	Rabbit polyclonal	Cell Signaling Technology
p-ERK1/2	1:1000	/	/	/	Rabbit polyclonal	Cell Signaling Technology
p38	1:1000	/	/	/	Rabbit polyclonal	Cell Signaling Technology
